# Structural and stability differences among GI norovirus virus-like particles produced in silkworm–baculovirus expression vector system

**DOI:** 10.3389/fmicb.2025.1739683

**Published:** 2026-01-08

**Authors:** Yuto Tsurumi, Akitsu Masuda, Jian Xu, Hiroaki Mon, Takahiro Kusakabe, Jae Man Lee

**Affiliations:** 1 Laboratory of Insect Genome Science, Graduate School of Bioresource and Bioenvironmental Sciences, Kyushu University, Fukuoka, Japan; 2 Laboratory of Creative Science for Insect Industries, Graduate School of Bioresource and Bioenvironmental Sciences, Kyushu University, Fukuoka, Japan; 3 Laboratory of Insect Food Science, Graduate School of Bioresource and Bioenvironmental Sciences, Kyushu University, Fukuoka, Japan

**Keywords:** norovirus, virus-like particles, vaccine development, silkworm–baculovirus expression system, structure

## Abstract

Norovirus (NoV) is a leading cause of acute gastroenteritis worldwide, with genogroups I and II (GI and GII) most frequently detected. NoV Virus-like particles (VLPs) composed of the major capsid protein VP1 (~60 kDa) are essential for vaccine development, yet GI VLPs remain poorly characterized. In this study, VP1 from four epidemiologically relevant GI genotypes was expressed using the silkworm-baculovirus system, and purification conditions were optimized in a genotype-specific manner. Purified VLPs were analyzed using size-exclusion chromatography (SEC), dynamic light scattering (DLS), transmission electron microscopy (TEM), and differential scanning fluorimetry (DSF), and their functional epitopes confirmed via binding to histo-blood group antigens (HBGAs) mimics. Stability assessments revealed genotype-dependent differences in pH and thermal tolerance, including aggregation and structural transitions. Collectively, these findings define genotype-specific purification, structural characterization, and stability profiles for four GI NoV VLPs. This work provides the first systematic framework for understanding GI-specific constraints in VLP production and offers foundational data that directly inform the design and formulation of multivalent NoV vaccines.

## 1 Introduction

Norovirus (NoV) is a globally present endemic that is the main cause of acute gastroenteritis (Morillo and Timenetsky, 2011; Flynn et al., 2024). The estimated annual mortality attributed to it ranges from 185,000 to 219,000, primarily affecting infants, the elderly, and populations in low- and middle-income countries (LMICs) (Harris et al., 2008; Pires et al., 2015; Grant et al., 2017; Platts-Mills et al., 2015). The economic impact is also substantial, with annual losses in the United States alone over USD 6 billion (Bartsch et al., 2016). To date, however, no vaccines or specialized antiviral therapies have been approved (Glass et al., 2009; Netzler et al., 2019).

NoV is a non-enveloped, positive-sense, single-stranded RNA virus within the *Caliciviridae* family (Robilotti et al., 2015). It exhibits significant genetic diversity and is currently categorized into 10 genogroups (GI–GX), with further subdivisions into multiple genotypes (e.g., GII.4) (Chhabra et al., 2019; Kendra et al., 2022). GI and GII genogroups are the most frequently identified among infected populations, while the prevalent genotypes change across years and geographical areas (de Graaf et al., 2016; Cannon et al., 2021). The NoV genome (~7.5 kbp) contains three open reading frames (ORF1–3) (Morillo and Timenetsky, 2011). ORF1 codes for non-structural proteins that are indispensable for viral replication, while ORF2 codes for the main capsid protein VP1, and ORF3 encodes the minor structural protein VP2 (Robilotti et al., 2015). The *T* = 3 icosahedral capsid (~40 nm in diameter) is assembled mainly from 180 VP1 monomers, with VP2 contributing to structural stability (Taniguchi et al., 1981; Bertolotti-Ciarlet et al., 2003; Vongpunsawad et al., 2013). VP1 consists of two major structural domains: the shell (S) domain, which forms the contiguous icosahedral shell, and the protruding (P) domain, which extends outward from the shell (Prasad et al., 1999; Jung et al., 2019). The P domain can be further divided into the P1 (located adjacent to the S domain) and the distal P2 subdomains (Hu et al., 2022). The histo-blood group antigens (HBGAs) binding site, essential for viral attachment and entry, is located within the P2 subdomain (Huang et al., 2005; Shirato et al., 2008; Uusi-Kerttula et al., 2014). In addition, the P2 subdomain harbors other host interaction sites, although this region exhibits relatively low amino acid sequence conservation among reported genotypes (Tan et al., 2009; Ford-Siltz et al., 2022; Kimura-Someya et al., 2024).

To date, NoV has been successfully propagated in human intestinal enteroids and in human epithelial cells differentiated from induced pluripotent stem cells (iPSCs) (Jones et al., 2014; Ettayebi et al., 2016; Estes et al., 2019; Sato et al., 2019; Hayashi et al., 2024). Nevertheless, efficient and reproducible viral culture remains technically challenging. As a result, virus-like particles (VLPs), which structurally mimic the viral capsid but lack the viral genome, are widely employed as surrogate models for NoV research and vaccine development. NoV VLPs, with diameters of approximately 40 nm, closely resemble native virions and compose 180 VP1 subunits assembled into a capsid (Jiang et al., 1992; Pogan et al., 2018a). Purified VLPs serve as important tools to study viral entry, capsid structure, host interactions, and vaccine candidates (Garaicoechea et al., 2015; Nelson et al., 2018; Esposito and Principi, 2020; Hu et al., 2022; Ayyar et al., 2023; Hadj Hassine et al., 2024). However, most studies have primarily focused on VLPs derived from GI.1 (prototype Norwalk virus) and GII.4 (the globally predominant genotype) VLPs, whereas other genotypes are relatively less studied.

The silkworm–baculovirus expression vector system (Silkworm-BEVS) is a high-yield expression system for heterologous proteins (Hitchman et al., 2011; Qi et al., 2016; Ebihara et al., 2021; Kusakabe, 2023). In our previous study, while GII-derived VLPs were efficiently purified, exhibited high solubility, and generated well-defined size-exclusion chromatography (SEC) profiles corresponding to uniform VLP particles, GI-derived VLPs exhibited pronounced genotype-specific limitations. Specifically, GI.2 required trehalose to maintain solubility, GI.3 and GI.6 showed intrinsically low solubility, with GI.6 being almost insoluble, and GI.4 formed structurally heterogeneous particles during assembly (Tsurumi et al., 2025). Consequently, the yield and purity of GI VLPs were consistently inferior to those of GII VLPs, and their structural and biochemical properties remain largely undefined.

Based on these observations, we hypothesized that the poor productivity and instability of GI VLPs arise from genotype-dependent differences in VP1 folding, dimer stabilization, and higher-order assembly mechanisms that fundamentally differ from those of GII VLPs. We further hypothesized that systematic optimization of solubilization buffers and purification conditions could partially overcome these limitations and reveal latent genotype-specific differences in particle stability, particularly for GI.4. Accordingly, the present study was designed to (i) define the specific biochemical obstacles associated with each GI genotype, (ii) clarify whether GI VLPs exhibit predictable genotype-dependent differences in solubility and stability, and (iii) test the hypothesis that GI VLP instability originates from intrinsic VP1 assembly properties rather than from expression yield alone.

Here, we report a genotype-specific purification strategy for GI NoV VLPs, optimizing solubilization buffer pH, sucrose cushion conditions, and anion exchange chromatography. Purified VLPs were characterized by SEC, DLS, and TEM, and functional epitopes confirmed by HBGA binding (Haynes et al., 2019). Thermal and pH stability analyses revealed genotype-dependent differences and stepwise structural transitions from aggregation to capsid collapse. Overall, this study established the genotype-specific purification parameters and stability profiles of GI NoV VLPs, providing useful information for the NoV VLP-based vaccine development in the future.

## 2 Materials and methods

### 2.1 Cell culture and silkworm strain

BmN cells (Funakoshi Co., Ltd., Tokyo, Japan) were maintained in 4 mL culture flasks (Falcon, Corning Inc., Corning, NY, USA) at 27 °C in IPL-41 insect culture medium (Sigma-Aldrich, St. Louis, MO, USA) supplemented with 10% fetal bovine serum (FBS; Gibco, Grand Island, NY, USA). The silkworm strain d17, provided by the Genetic Resource Research Institute, Kyushu University, was used in this study. Larvae were reared on mulberry leaves at 24–29 °C.

#### 2.2 Generation of recombinant baculovirus

The DNA sequences encoding ORF2 (VP1) from NoV GI.2 (GenBank accession no. AAA92984), GI.3 (GenBank accession no. QHR93636), GI.4 (GenBank accession no. BAB18267), and GI.6 (GenBank accession no. AAC64603) were used. Recombinant baculoviruses were generated as described previously (Tsurumi et al., 2025). Briefly, each VP1 gene was cloned between the polyhedrin promoter and the SV40 polyadenylation signal in the pFastBac vector (Invitrogen, Carlsbad, CA, USA) using the NEBuilder Golden Gate Assembly Kit (New England Biolabs, Ipswich, MA, USA) to construct transfer plasmids. These transfer plasmids were used to generate recombinant BmNPV T3^Δsix-gene^ bacmids, in which six non-essential genes (*egt*, *chitinase*, *v-cathepsin*, *p26*, *p10*, and *p74*) were deleted to prevent unnecessary VP1 cleavages (Ebihara et al., 2024; Tsurumi et al., 2025). For transfection, BmN cells were seeded in 24-well plates (Falcon; Corning Inc., Corning, NY, USA) at approximately 70–80% confluence. Bacmid DNA (10 μL dissolved in ddH2O) was mixed with 1 μL Avalanche-Everyday reagent (Oz Biosciences, Marseille, France), adjusted to a final volume of 50 μL with serum-free KBM720 medium (Kohjin Bio, Saitama, Japan), and incubated at room temperature for 15 min. The transfection mixture was then added to the cells. Recombinant baculoviruses (P1) produced by transfected cells were collected and applied for serial infections to generate high-titer P3 viral stocks, which were filtered through a 0.2 μm Millex-LG filter (Merck Millipore, Burlington, MA, USA) and stored at 4 °C until use. These viral stocks were subsequently injected into silkworm pupae for VP1 protein expressions.

#### 2.3 Expression and extraction of NoV VP1

The recombinant virus (BmNPV T3^Δsix-gene^ /NoV VP1) was injected into d17 silkworm pupae at a dose of 1 × 10^4^ plaque-forming units (pfu) per pupa. Pupae infected with the recombinant virus were incubated at 27 °C, collected 8 days post-infection (d.p.i.), and stored at −80 °C until use. For protein extraction, three frozen pupae infected with BmNPV/NoV VP1 were thawed and homogenized in 5 mL of extraction buffer per pupa (50 mM sodium phosphate buffer, 500 mM NaCl, pH 6.0, 7.0, or 8.0). The homogenate was sonicated on ice for 10 min using a Tomy UD-100 sonicator (Tomy Seiko, Tokyo, Japan) and subsequently centrifuged at 11,000×*g* for 30 min at 4 °C. The supernatants were collected, and the pellets were resuspended in an equal volume of the corresponding extraction buffer. The expression levels and solubility of VP1 in the silkworm pupae were assessed by Coomassie Brilliant Blue (CBB) staining following 10% SDS-PAGE.

### 2.4 Optimizations of the purification process of NoV VP1

#### 2.4.1 Sucrose cushion ultracentrifugation

Supernatants from three silkworm pupae were pooled and subjected to sucrose cushion ultracentrifugation for VP1 purification. Briefly, 15 mL of clarified supernatant was layered onto a two-step sucrose cushion consisting of 10 mL each of 30% (w/v) and 60% (w/v) sucrose. Samples were centrifuged at 100,000×*g* for 4 h at 4 °C. Following ultracentrifugation, fractions were collected, and the presence of VP1 was confirmed by SDS-PAGE and CBB staining. The resulting insoluble pellet was resuspended in 1 mL of sodium phosphate buffer (pH 7.0) containing 500 mM NaCl and subjected to further analyses. VP1-containing fractions were dialyzed overnight at 4 °C against sodium phosphate buffer (pH 7.0) to remove sucrose. Dialysis was performed using a membrane with a molecular weight cut-off (MWCO) of 14 kDa (Fisher Scientific, Waltham, MA, USA) without stirring.

#### 2.4.2 Anion exchange chromatography

Anion exchange chromatography was performed using an ÄKTA avant 25 system (Cytiva, Tokyo, Japan). Samples were applied either to a 1 mL CIMmultus™ QA columns (Sartorius, Göttingen, Germany) or 5 mL HiTrap™ Q HP columns (Cytiva, Tokyo, Japan). After sample loading, the columns were washed with 5–10 column volumes (CV) of binding buffer (50 mM sodium phosphate, pH 7.0) at room temperature, and bound proteins were eluted at a flow rate of 5 mL/min using a linear gradient of 0–1 M NaCl. For GI.2 and GI.4, three fractions corresponding to major elution peaks were collected and subjected to subsequent analyses. For GI.3, stepwise elution was employed; the washing buffer contained 125 mM NaCl in 50 mM sodium phosphate, and the elution buffer contained 450 mM NaCl in 50 mM sodium phosphate. Three peak fractions obtained by stepwise elution were collected for further analysis. All collected fractions were evaluated by SDS-PAGE to confirm the presence and purity of VP1.

### 2.5 Characterization of purified VP1 proteins

#### 2.5.1 Dynamic light scattering (DLS)

The size distribution of the NoV VP1 protein was analyzed by dynamic light scattering (DLS) using an ELSZ-2000ZS instrument (Otsuka Electronics Co., Ltd., Osaka, Japan). Measurements were performed at room temperature. DLS analyses were conducted in two independent experiments, each comprising three technical replicates. Because these replicates do not represent independent biological samples, the results are presented descriptively without formal statistical testing.

#### 2.5.2 Size exclusion chromatography (SEC)

Size exclusion chromatography (SEC) was performed on an ÄKTA avant 25 system (Cytiva, Tokyo, Japan) using a Superose™ 6 Increase 10/300 GL column (Cytiva, Tokyo, Japan). The column was equilibrated and eluted with 50 mM sodium phosphate buffer containing 500 mM NaCl (pH 7.0) at room temperature. Protein fractions obtained from SEC were analyzed by SDS-PAGE to assess purity and estimate molecular size. The tailing factor (TF) of SEC chromatograms was calculated using the elution peak of the resolved fraction. Chromatogram data (absorbance vs. time) were imported into Microsoft Excel (Microsoft 365), and the peak height was determined using the MAX function. The times corresponding to 5% of the peak height on the left and right sides of the peak were obtained using the INDEX and MATCH functions. The tailing factor was calculated using the following equation:


$$\mathrm{TF}=\frac{t_{\text{right}}-t_{\text{left}}\;}{2\times (\;t_{\text{peak}}-t_{\text{left}}\;)}$$

where t_peak_ is the time at the peak apex, t_left_ is the time at 5% of the peak height on the ascending side, and t_right_ is the time at 5% of the peak height on the descending side. If the signal did not reach 5% after the peak, the last data point of the post-peak region was used.

#### 2.5.3 Transmission electron microscopy (TEM)

Based on a previous study (Masuda et al., 2023), recombinant baculoviruses in samples collected from silkworm pupae were inactivated with binary ethyleneimine at 37 °C for 24 h before the purification process described above for TEM analysis. The purified VP1 protein was adsorbed onto carbon-coated copper grids (Oken Shoji, Tokyo, Japan) at room temperature. Excess liquid was gently removed with filter paper, and the sample was stained with 2% (w/v) uranyl acetate. Residual staining solution was blotted at the grid edge and air-dried. The specimens were observed using a Tecnai 20 transmission electron microscope (FEI Company, Hillsboro, OR, USA) under 50,000× or 80,000× magnification and operating conditions.

### 2.6 PGM binding assay

NoVs can interact with human histo-blood group antigens (HBGAs), which play a crucial role in viral attachment and infection. The pig gastric mucin (PGM), which contains multiple HBGA types similar to those found in human saliva, is widely used to evaluate the HBGA-binding activity of NoV VLPs (Tan et al., 2004). The PGM-binding assay was performed with slight modifications of the previously described protocols (Masuda et al., 2023). Briefly, 96-well flat-bottom MaxiSorp plates (Thermo Fisher Scientific) were coated with 5 μg/mL PGM (Sigma-Aldrich, St. Louis, MO, USA) and incubated overnight at 4 °C. Plates were blocked with StartingBlock™ Blocking Buffer at 37 °C for 1 h. Purified NoV VLPs were diluted in genotype-specific buffers based on previously reported conditions: GI.2 and GI.4 VLPs were diluted in 50 mM sodium phosphate buffer (pH 6.5) containing 500 mM NaCl, and GI.3 VLPs were diluted in 50 mM sodium phosphate buffer (pH 7.5) containing 500 mM NaCl (Haynes et al., 2019). Diluted VLPs were added to the wells and incubated at 37 °C for 1 h. After washing with PBST (PBS containing 0.05% Tween-20), wells were incubated with anti-norovirus GI.1 monoclonal antibody (B1928M, Abcam, Cambridge, UK) diluted 1:1000 in StartingBlock™ blocking buffer containing 0.05% Tween-20 for 1 h at 37 °C. Following additional washes with PBST, HRP-conjugated goat F (ab′) anti-mouse IgG H&L (ab6823, Abcam, Cambridge, UK) diluted 1:15,000 in the same blocking buffer was added and incubated at 37 °C for 1 h. After the final wash, TMB substrate (Thermo Fisher Scientific) was added for color development. The reaction was stopped with 2 M H_2_SO_4_, and absorbance was measured at 450 nm with reference 620 nm using a multimode microplate reader (Nivo, PerkinElmer, Waltham, MA, USA) and MyAssays Desktop Standard software. Optical density (OD) values were calculated by subtracting 620 nm reference and the blank control from the absorbance at 450 nm. The assay was performed in three independent experiments, each with three technical replicates.

### 2.7 Stability assay of purified NoV VLPs

#### 2.7.1 pH stability assay

The pH stability of NoV VLPs was evaluated using the GeBAflex-tube Mini Dialysis Kit (MWCO 6–8 kDa; Funakoshi, Tokyo, Japan). VLPs were dialyzed overnight at 4 °C against buffers containing 500 mM NaCl, each adjusted to a different pH: 50 mM acetate buffer (pH 3.0, 4.0, or 5.0), 50 mM phosphate buffer (pH 6.0, 7.0, or 8.0), or 50 mM Tris–HCl buffer (pH 9.0). Following dialysis, the hydrodynamic diameter of VLPs was determined by DLS as described above. pH stability analysis by DLS was performed in two independent experiments, with three technical replicates per condition.

For TEM, residual baculovirus in the samples was inactivated by BEI as described above, and the dialyzed VLPs were subjected to the same pH conditions used for the DLS measurements.

#### 2.7.2 Thermal stability assay

Differential scanning fluorimetry (DSF) was used to evaluate the thermal stability of NoV VLPs. For this assay, VLP samples were further purified by size-exclusion chromatography following anion-exchange chromatography to ensure higher purity. Purified NoV VLPs were adjusted to a final concentration of 0.5 mg/mL and mixed with SYPRO™ Orange Protein Gel Stain (Invitrogen, Carlsbad, CA, USA) at a final 20× concentration. A total of 20 μL of the mixture was dispensed into each well of a 96-well PCR plate, and the plate was heated from 25 °C to 95 °C at a rate of 1 °C per minute. Fluorescence was monitored using the Applied Biosystems StepOneplus™ Real-Time PCR System, with detection in the ROX channel (excitation/emission: 470/570 nm). Melting curves and apparent melting temperatures (Tm values) were determined using the DSFworld software.^1^ Thermal stability analysis was performed independently for three times, with each experiment including three technical replicates.

### 2.8 SDS-page

All protein samples were mixed with an equal volume of 2× SDS sample buffer (0.1 M Tris–HCl, pH 6.8; 0.2 M dithiothreitol; 4% SDS; 20% glycerol; 0.02% bromophenol blue) and denatured at 95 °C for 10 min. The denatured samples were separated on 10% SDS-PAGE and visualized by Coomassie Brilliant Blue (CBB) staining. The molecular weight marker used for SDS-PAGE was prepared by mixing the following purified proteins: T7 RNA polymerase (100 kDa), bovine serum albumin (66 kDa), glutamate dehydrogenase (55.6 kDa), glyceraldehyde-3-phosphate dehydrogenase (37 kDa), l-lactate dehydrogenase (36.5 kDa), carbonic anhydrase (29 kDa), and lysozyme (14.4 kDa).

### 2.9 Statistical analysis

For the thermal stability data, the normality of distributions was assessed by the Shapiro–Wilk test. Differences among genotypes were analyzed using the Kruskal–Wallis test, followed by *post hoc* pairwise comparisons with Dunn’s test, applying Holm’s correction for multiple comparisons. A *p*-value below 0.05 was considered statistically significant. All statistical analyses were performed in R version 4.3.0.

## 3 Results

### 3.1 Effect of extraction pH on VP1 solubility

**Figure 1:**
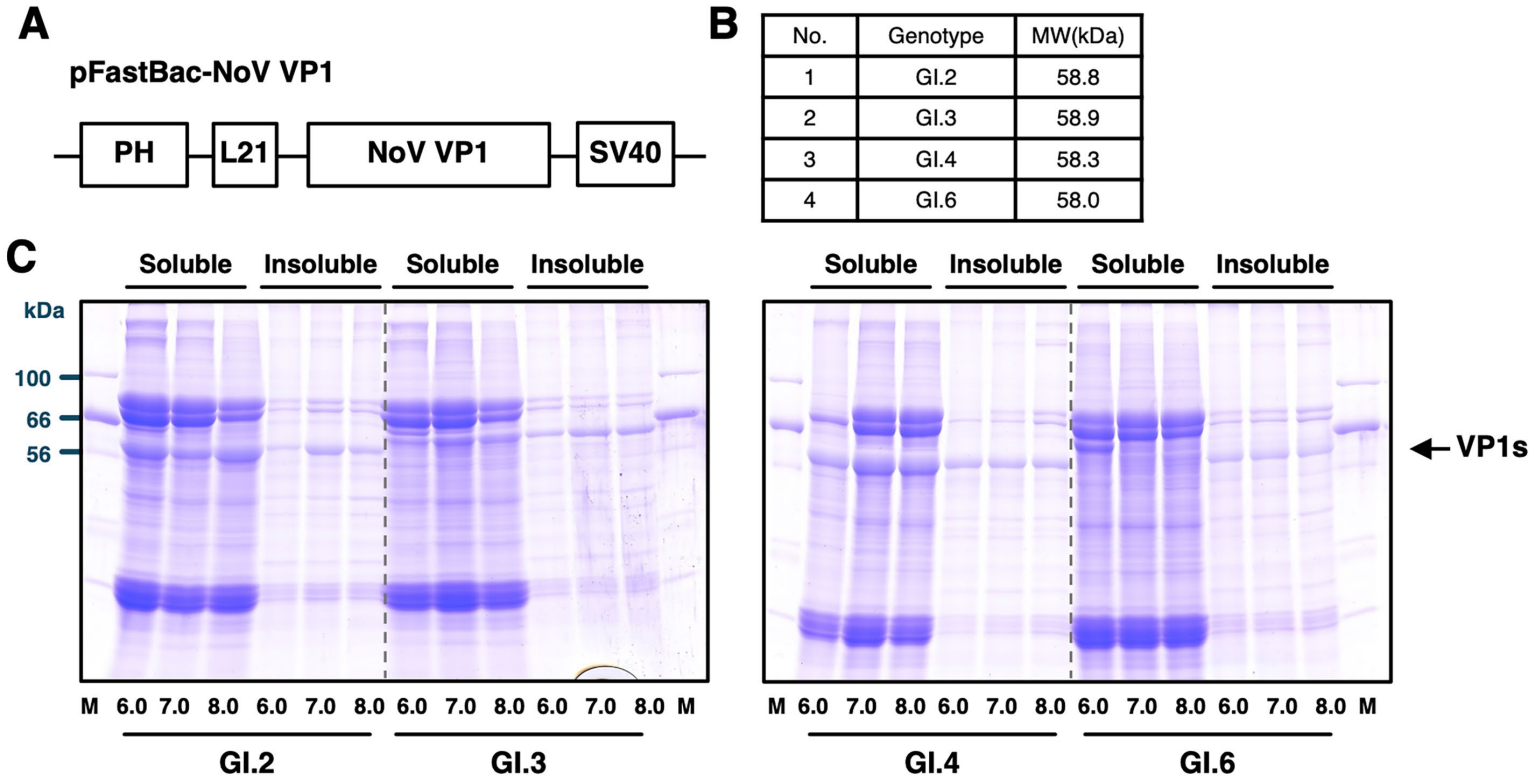
Construction of the VP1 expression cassette and evaluation of pH-dependent solubility. **(A)** Schematic representation of the VP1 expression cassette. The ORF2 region of the norovirus (NoV) genome is positioned downstream of the polyhedrin promoter (PH) and terminated by the SV40 polyadenylation signal. An L21 sequence derived from lobster tropomyosin cDNA is inserted upstream of ORF2 to enhance translation efficiency. **(B)** Predicted molecular weights of the NoV VP1 proteins. **(C)** Effect of buffer pH on the solubility of VP1 derived from GI.2, GI.3, GI.4, and GI.6. Crude extracts prepared from three infected pupae were centrifuged to separate the soluble and insoluble fractions, which were analyzed by 10% SDS-PAGE. The position of VP1 is indicated by arrows on the right.

NoV VP1 was expressed using a construct identical to that described in previous studies (Tsurumi et al., 2025) (Figure 1A) via the Silkworm-BEVS. To avoid unexpected proteolytic cleavage of expressed VP1 proteins, the recombinant baculovirus lacking several baculovirus-derived proteases (T3^Δsix-gene^) was employed in this study, as described previously (Tsurumi et al., 2025). As listed in Figure 1B, the expected molecular weights of VP1s (GI.2, 3, 4 and 6) are approximately 58–59 kDa. Previous studies on GI genogroup viruses demonstrated that varying the extraction buffer pH can solubilize VP1 from insect cells (Kimura-Someya et al., 2024). In addition, studies on murine NoV reported that acidic conditions stabilize the particle structure (Sherman et al., 2024). Based on these findings, we investigated how extraction buffer pH affects VP1 solubility. As shown in Figure 1C, GI.2 showed the highest solubility at pH 6.0. The solubility decreased when the buffer was adjusted to pH 7.0, and an unexpected increase was observed again at pH 8.0. GI.3 showed maintained approximately 50% solubility across all tested pH values, which suggests that this genotype has inherently low extractability. GI.4 exhibited poor solubility at pH 6.0 but improved solubility at both pH 7.0 and 8.0. This pattern differs from earlier studies that reported more efficient extraction under acidic conditions. In contrast, GI.6 VP1 remained largely insoluble under all tested pH values (6.0–8.0), indicating that efficient recovery for GI.6 VP1 may require co-expression with additional viral proteins such as VP2 or the presence of stabilizing cofactors including metal ions (Hu et al., 2022). Taken together, these results showed that the solubility of GI VP1 proteins produced by Silkworm-BEVS exhibits a genotype-specific pH sensitivity.

Based on these findings, the buffer conditions were optimized for downstream purification. Phosphate buffer at pH 6.0 was applied for GI.2, while phosphate buffer at pH 7.0 was used for GI.3 and GI.4. Given the negligible solubility of GI.6 VP1 under all conditions examined, this genotype was then excluded from further purification and analysis in this study.

### 3.2 Purification by sucrose cushion and anion exchange chromatography

**Figure 2:**
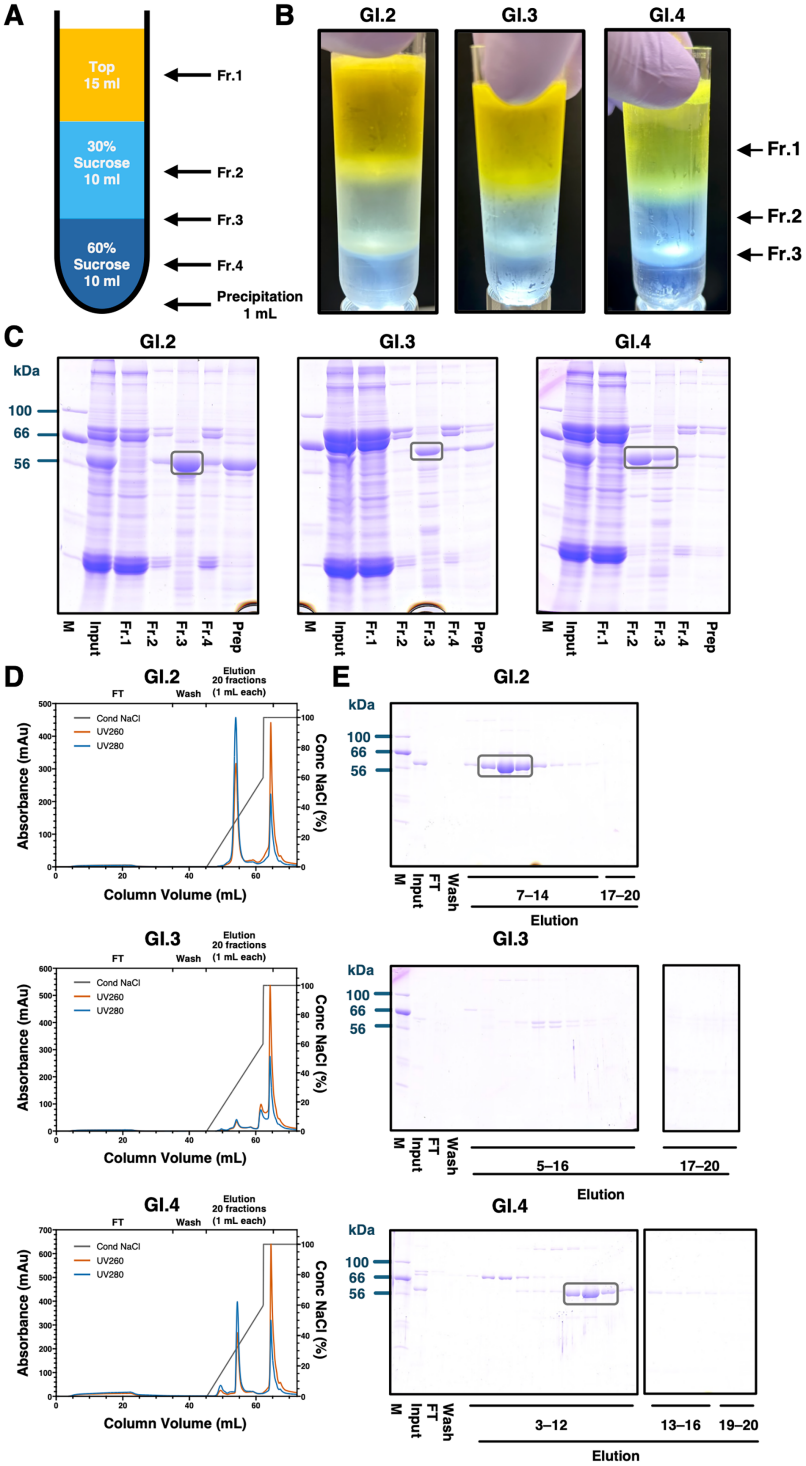
Purification of VP1 by sucrose cushion centrifugation and anion exchange chromatography. **(A)** Schematic overview of the sucrose cushion setup. The precipitate was recovered by resuspending it in 1 mL of sodium phosphate buffer containing 500 mM NaCl (pH 7.0). **(B)** Photograph of the centrifuge tube after ultracentrifugation (*n* = 3). Fr.1 indicates the top of the sucrose cushion, Fr.2 the 30% sucrose layer, and Fr.3 the interface between the 30 and 60% sucrose layers, each marked by arrows. **(C)** Analysis of each fraction collected from the sucrose cushion by 10% SDS-PAGE. The position of VP1 in the lanes of the collected fractions is indicated by a black box. **(D)** Anion exchange chromatogram obtained using a monolith-type anion exchange column (*n* = 3). NaCl concentration is shown in gray, UV absorbance at 260 nm in red, and absorbance at 280 nm in blue. The collection points for the flow-through (FT), wash, and elution fractions are indicated at the top of the graph. Elution was performed using a linear gradient from 0 to 60% NaCl, collecting 18 fractions (1 mL each), followed by 2 fractions with 100% NaCl, yielding a total of 20 fractions. **(E)** 10% SDS-PAGE analysis of fractions corresponding to elution peaks in the chromatogram. Lane numbers correspond to the elution fractions collected as indicated in panel D. The position of VP1 in the lanes of the collected fractions is indicated by a black box.

Sucrose cushion ultracentrifugation is widely used for the purification of high-molecular-weight complexes such as VLPs. In our previous study, GII.4 VP1 was successfully purified to a high degree of purity using a two-layer sucrose cushion consisting of 30 and 60% sucrose (Masuda et al., 2023). Following this approach, we applied the same method for the initial purification of GI VLPs. VLPs were expected to accumulate at the interface between the two layers, corresponding to Fr.3 in Figure 2A. Ultracentrifugation resulted in the formation of a visible intermediate layer (Fr.3, Figure 2B). Subsequently, each layer (Frs. 1–3) was collected and analyzed by 10% SDS-PAGE. As shown in Figure 2C, VP1 from GI.2 and GI.3 was enriched in the intermediate layer (Fr. 3), whereas GI.4 VP1 was mainly detected in the 30% sucrose layer (Fr.2). In sucrose ultracentrifugation, particle migration is determined by density, size, and shape. When immature or denatured forms are present, or when proper particle assembly is not maintained, equilibrium may be reached in the 30% sucrose layer. The absence of GI.4 VP1 accumulation in the intermediate layer was presumed to reflect partial dissociation in the aqueous sucrose solution. To minimize such destabilization, the solvent of the 30% sucrose layer was further replaced with sodium phosphate buffer for the GI.4 VP1 sample. This adjustment led to an improved recovery of GI.4 VP1 in the intermediate layer (Supplementary Figure 1A), confirming its sensitivity to aqueous sucrose conditions. Based on these findings, VP1s from GI.2 and GI.3 were recovered from the 30% / 60% interface, while GI.4 VP1 was collected from both the 30% layer and the interface.

To remove host-derived proteins, nucleic acid, and other contaminants that not eliminated by sucrose cushion centrifugation, anion-exchange chromatography was then performed. Our previous research employed a HiTrapTM Q HP column (Tsurumi et al., 2025), but it was ineffective due to insufficient VP1 binding and inadequate host protein separation. To address this, we tested a monolith-type column, which provides higher surface accessibility and binding capacity. As shown in Figures 2D,E, whereas HiTrap™ Q HP required genotype-specific salt concentrations and produced broad elution profiles, the monolith-type anion exchange column yielded sharper peaks within the 25–40% NaCl range, significantly enhancing both yield and purity. This improvement was particularly noticeable in GI.4, which exhibited greater host protein contamination and weaker retention on the HiTrap™ Q HP column (Supplementary Figures 1B,C). With strong absorbance at 260 nm, a late-eluting peak at 1 M NaCl was found to be nucleic acid, which is consistent with earlier studies (Masuda et al., 2023), indicating that the column successfully removed nucleic acids. In the case of GI.3, VP1 showed partial fragmentation under linear NaCl gradient elution, but this was effectively prevented by using a fixed 45% NaCl elution condition. Fractions obtained under this optimized condition were subsequently used for the downstream experiments (Supplementary Figures 1D,E).

### 3.3 Assessment of VLP formation and PGM binding

**Figure 3:**
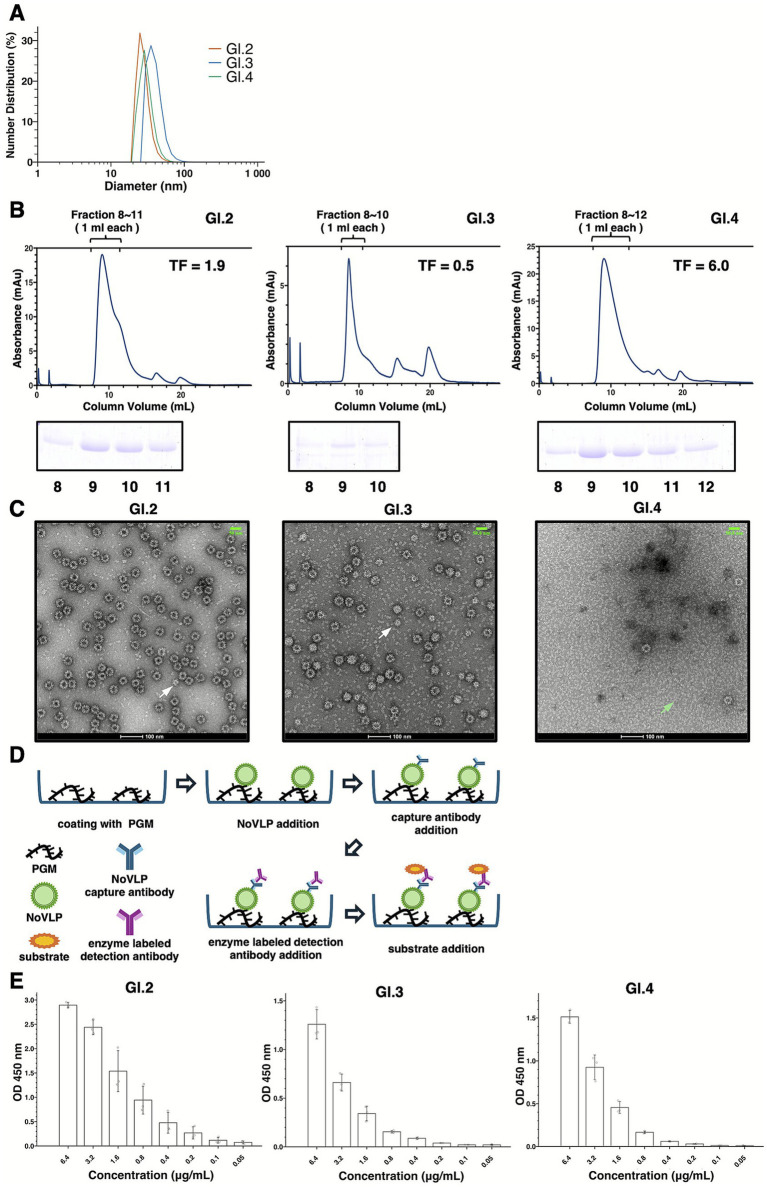
Characterization of purified VP1 particles and PGM-binding assay. **(A)** Particle size distribution of purified VP1 measured by dynamic light scattering (DLS), (*n* = 3). Data represent the average of three measurements. Results for GI.2, GI.3, and GI.4 are shown in red, blue, and green, respectively. **(B)** Size exclusion chromatography profile of purified VP1 (top). Peak elution fractions indicated above the chromatogram were collected and analyzed by 10% SDS-PAGE to confirm the presence of VP1 (bottom). Lane numbers correspond to the elution fractions indicated in the chromatogram. TF indicates the tailing factor of the chromatography. **(C)** Representative transmission electron microscopy (TEM) image of negatively stained VP1 particles. Five images each were taken at ×50,000 and ×80,000 magnification, and one representative image is shown. White arrows indicate small particles, and green arrows indicate dissociated VLP components. The white scale bar at the bottom represents 100 nm, and the green scale bar at the upper right represents 50 nm. **(D)** Schematic representation of the PGM binding assay. NoV VLPs are incubated with porcine gastric mucin (PGM) immobilized on a plate. After washing, a primary antibody specific for VLP is added, followed by a peroxidase-conjugated secondary antibody. Finally, fluorescence intensity is measured as the assay readout. **(E)** Porcine gastric mucin (PGM)-binding assay results (*n* = 3). Absorbance at 450 nm (OD450) was calculated by subtracting the values at 620 nm and blank. Experiments were performed in triplicate, and error bars represent the standard deviation.

VLP formation was then evaluated using dynamic light scattering (DLS), size-exclusion chromatography (SEC), and negative-stain transmission electron microscopy (TEM). Particle size was initially assessed by DLS (*n* = 3), which revealed genotype-specific differences in hydrodynamic diameter (D50). Particles with mean D50 values of ~24.74 nm (GI.2), ~34.84 nm (GI.3), and ~26.86 nm (GI.4) were detected (Figure 3A, Supplementary Table 1). Previous studies have reported genotype-dependent size variations in VLPs (Pogan et al., 2018a; Jung et al., 2019), and the observed differences in D50 are consistent with this trend. The SEC profiles resembled those previously reported for GII.4 VLPs (Masuda et al., 2023; Tsurumi et al., 2025), showing distinct elution volumes and peak shapes (Figure 3B). However, all GI genotypes exhibited peak tailing, which was most prominent in GI.4. While sharp peaks were observed for GII VLPs, the peak tailing of GI genotypes in this study suggests that GI VLPs could be more prone to dissociate or display greater heterogeneity in particle assembly. To visualize the possible heterogeneity, TEM was then employed to assess the VLP particle morphology. As shown in Figure 3C, approximately 30–40 nm particles were examined across all genotypes. GI.2 consisted mainly of 30–40 nm particles, although a few smaller particles were also observed. GI.3 exhibited both 30–40 nm particles and smaller particles less than 30 nm in diameter. In contrast, GI.4 produced relatively few VLPs, with many VP1-like structures lacking proper assembly. Collectively, these results indicate that although purified GI VP1 formed VLP particles with the expected size of 30–40 nm (Figures 3A,C), the GI genotypes displayed marked heterogeneity in particle morphology (Figure 3C), consistent with the peak tailing observed in SEC (Figure 3B). Furthermore, in light of the pH stability data (see Section 3.4), the acidic uranyl acetate staining solution likely contributed to the structural collapse of the GI.4 VLPs, potentially reflecting their instability at low pH (pH 3–5).

While DLS, SEC, and TEM analyses confirmed that purified VP1 assembled into particles, it remains essential to determine whether these particles preserve functional structural features, such as receptor-binding epitopes, to validate their applicability for epidemiological and pathological investigations. To address this, a porcine gastric mucin (PGM)-binding assay was performed (Figure 3D). The PGM binding assay demonstrated that all three genotypes retained PGM-binding activity (Figure 3E). Although previous studies have reported that certain GI.3 VLPs lack HBGA binding capacity (Wang et al., 2022), the GI.3 VLPs examined here clearly exhibited binding activity. It should be noted, however, that differences in antibody detection sensitivity among GI.2, GI.3, and GI.4 preclude a quantitative comparison of binding affinities (data not shown). Taken together, these results confirm that the purified VP1s assemble into ~30–40 nm VLPs which, despite some variation in size and morphology, maintain the functional epitopes required for receptor-mimetic binding.

### 3.4 Evaluation of VLP stability

As VLP vaccine candidates, it is essential to evaluate their susceptibility to physiological conditions such as pH fluctuations within the host. In addition, assessing their resistance to environmental stresses, including pH and temperature, is critical for determining the optimal storage conditions for manufactured vaccines. Despite this importance, basic data on the inactivation conditions of GI genogroup NoVs and the applicability of their VLPs as noninfectious surrogates remain limited. To address this gap, we profiled the resistance of purified GI VLPs to both pH and thermal stress.

**Figure 4:**
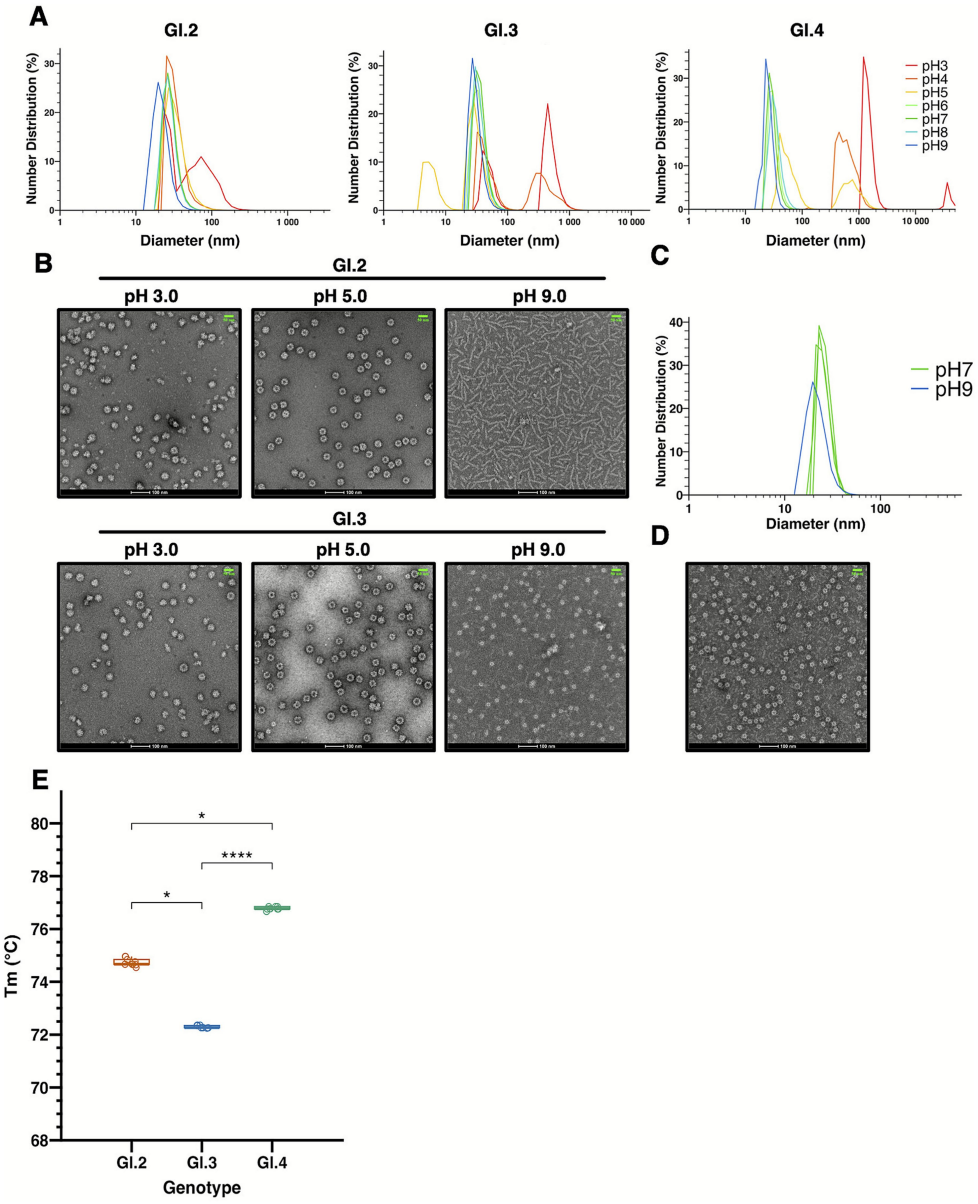
Evaluation of pH and thermal stability of VLPs. **(A)** Particle size distribution of VLPs after dialysis in buffers ranging from pH 3 to 9, measured by DLS (*n* = 3). Each color corresponds to the pH condition indicated in the legend. Data represent the average of three measurements per pH. **(B)** Representative TEM image of pH-treated samples. Samples were dialyzed overnight against buffers at each pH on the day before observation. They were then adjusted to a concentration of 0.1 mg/mL and stored at 4 °C until imaging. Five images were acquired at ×50,000 magnification, and one representative image is shown. The white scale bar at the bottom represents 100 nm, and the green scale bar at the upper right represents 50 nm. **(C)** Particle size measurement of GI.2 VLP dialyzed at pH 9.0 and subsequently exchanged into pH 7.0 buffer, analyzed by DLS (*n* = 3). Results of three replicates after dialysis are shown in green, while results at pH 9.0 are shown in blue. **(D)** TEM images of GI.2 VLP after dialysis at pH 9.0 and buffer exchange to pH 7.0. Five images were acquired at ×50,000 magnification, and one representative image is shown. The white scale bar at the bottom represents 100 nm, and the green scale bar at the upper right represents 50 nm. **(E)** Thermal denaturation profiles measured by differential scanning fluorimetry (DSF). Each measurement was performed in triplicate, and the entire experiment was repeated three times (*n* = 9). Error bars represent standard deviation. Statistical significance is indicated as follows: *p* < 0.05 (*), *p* < 0.0001 (****).

For pH stability, VLPs were dialyzed in buffers with different pH values ranging from 3.0 to 9.0 and further applied to DLS analysis (*n* = 3) (Figure 4A, Supplementary Tables 2–4). The results showed that GI.2 remained stable at pH 4.0 with a size distribution of 30–40 nm, but aggregated at pH 3.0, increasing in size to 50–100 nm. At pH 9.0, the particle size decreased to 20–30 nm. GI.3 exhibited partial aggregation or disassembly between pH 3.0 and 5.0, and a reduction of approximately 4 nm in D50 at pH 9.0. GI.4 was stable between pH 6.0 and 8.0 but aggregated at pH < 5.0 (particle size >500 nm) and decreased to 20–30 nm at pH 9.0. These divergent behaviors are indicative of genotype-specific variations in pH sensitivity, aligning with their respective extraction buffer preferences (Figure 1C). Negative-stain TEM further confirmed these findings for GI.2 and GI.3. After dialysis at pH 3.0, 5.0, and 9.0 TEM images revealed that GI.2 formed aggregates at pH 3.0, 30 ~ 40-nm hollow particles at pH 5.0, and sheet-like structures at pH 9.0 (Figure 4B). This latter observation indicates that the size reduction detected by DLS at alkaline pH may be caused by structural rearrangement rather than VLP disassembly.

For GI.3, TEM results confirmed a predominance of structurally compromised particles at pH 3.0, 30–40 nm particles at pH 5.0, and relatively smaller particles (~20-nm) at pH 9.0, which are consistent with the D50 reduction observed by DLS (Figure 4A). These small particles are likely *T* = 1 particles (White et al., 1997; Huo et al., 2015). Although less frequent than in GI.2, sheet-like structures were also detected in GI.3. To determine whether the structural changes of GI.2 VP1 at pH 9.0 are reversible, samples were re-dialyzed into pH 7.0 buffer and further analyzed by DLS and TEM. As shown in Figure 4C, DLS revealed a slight increase in particle size, suggesting a possible transition from fibrous to particulate structures (Supplementary Table 5). The TEM results validated the presence of a significant population of ~30 nm particles, presumably corresponding to *T* = 1 assemblies, along with 30–40 nm particles (Figure 4D). A few fibrous structures also persisted, indicating a mixture of small particles, native-sized particles, and residual fibrous assemblies.

The thermal stability of all three GI NoV VLPs was profiled using Differential Scanning Fluorimetry (DSF) to determine the structural collapse temperature of VP1 (Figure 4E, Supplementary Figure 2, Table 1). GI.2 exhibited thermal denaturation at approximately 75 °C, GI.3 at 72 °C, and GI.4 at 77 °C, indicating genotype-dependent differences in thermostability. Quantitative analysis (*n* = 9) confirmed the following order: GI.3 (72.30 °C ± 0.15) < GI.2 (74.75 °C ± 0.05) < GI.4 (76.78 °C, ±0.06).

**Table 1:** Heat stability evaluation using DSF.

Genotype	Tma (°C)	SD
GI.2	74.75 ± 0.049	0.146
GI.3	72.3 ± 0.016	0.049
GI.4	76.78 ± 0.02	0.059

## 4 Discussion

This study aimed to improve purification of GI VLPs, historically difficult to obtain in pure form, and to evaluate their genotype-specific structural and stability features. GI NoVs, despite pandemic potential, remain understudied, with limited reports on production or genotype-specific traits. Our previous work highlighted substantial diversity among GI VLPs, emphasizing the need for detailed characterization (Tsurumi et al., 2025). We present a rarely reported purification strategy and provide new insights into their stability and structural variations.

We first investigated the influence of buffer pH on VP1 solubilization, guided by previous reports suggesting that acidic conditions enhance solubility (Kimura-Someya et al., 2024). Consistent with previous studies, the most efficient solubilization of GI.2 occurred at pH 6.0 (Figure 1C). Solubilization at neutral pH (pH 7.0) was lower than at pH 8.0, contrary to previous reports indicating that VLPs are generally less stable under relatively basic conditions (Pogan et al., 2018a). GI.4 showed optimal solubility at neutral pH, with reduced efficiency at pH 6.0. Expression in silkworm-BEVS further reduced GI.4 solubility below pH 5.0, suggesting host-dependent modifications or subtle structural variations, consistent with previous reports (Devant and Hansman, 2021; Sherry et al., 2025). The consistently low solubility observed for GI.3 and GI.6 across pH conditions suggests these VP1 proteins may tend to aggregate or misfold, potentially influenced by host- or buffer-dependent factors such as VP2, divalent ions, or glycosylation. However, our study did not directly test the effects of metal ions, bile acids, or VP2 on GI.3 and GI.6 VLP solubility, and thus these proposed mechanisms remain speculative. In GII.4 and murine norovirus (MNV) VLPs, divalent metal ions stabilize dimer formation, bile acids contribute to P-domain stabilization, and VP2 enhances overall VLP stability (Sherman et al., 2019, 2024; Hu et al., 2022; Liao et al., 2022). It will be important to examine how these factors influence GI.3 and GI.6 solubility, and whether or not introducing human-like glycosylation affects VLP production (Taghizadeh et al., 2024).

As shown in Figures 2–4, the behavior of VP1s during purification and the properties of purified VLPs also differed among genotypes. GI.3 VP1 underwent cleavage during anion exchange chromatography, suggesting it is prone to misfolding. This misfolding may make it susceptible to proteolysis, consistent with its low solubility in the tested extraction buffers. GI.4 VLPs exhibited particle heterogeneity validated in both sucrose cushion ultracentrifugation and SEC. Previous work demonstrated that introducing disulfide mutations at the dimer interface enabled the production of uniform GI.1 VLPs (Verardi et al., 2020). Given the peak tailing observed in SEC, GI.4 VLPs may dissociate into dimers or higher-order oligomeric complexes. Mutations with stabilizing effects may therefore be required to generate functionally homogeneous particles and avoid inducing non-neutralizing antibody responses. Interestingly, GI.4 demonstrated elevated solubility compared to other tested GI genotypes, which suggests that VP1 solubility does not invariably correlate with assembled VLP stability.

PGM-binding assays further demonstrated that all three genotypes were capable of binding to receptor-mimetic molecules, although antibody sensitivity varied among genotypes and precluded precise comparisons. The development or acquisition of antibodies with equivalent detection efficiency across genotypes would allow accurate profiling of PGM-binding affinities, which in turn may help elucidate genotype-specific mechanisms of host attachment and infection.

Stability tests revealed clear genotype-specific differences in both pH and thermal stability. When considering VLPs as vaccine antigens, it is essential to predict how environmental changes *in vivo* may affect their structural integrity. Previous studies have reported pH-dependent stability for GII.4, GII.17, and GI.1. GI.1 maintains *T* = 3 particle formation at pH 6.0–7.0, transitions to *T* = 1 at pH 8.0, and loses particles at pH 9.0. One subtype maintains particles at pH 3.0–7.0 but forms *T* = 1 or disassembles at pH 8.0 (Ausar et al., 2006; da Silva et al., 2011; Pogan et al., 2018b). GII.4 remains stable at pH 3.0–8.0 (da Silva et al., 2011; Samandoulgou et al., 2015), whereas GII.17 maintains VLP particle formation between pH 6.0 and 10.0 (Pogan et al., 2018b). In this study, GI.2 exhibited relatively high resistance to acidic conditions, similar to GI.1, and maintained structural stability across a broad pH range (pH 4.0–8.0). At pH 9.0, GI.2 VP1 transitioned from spherical to tubular structures. These may represent intermediate-like states in VLP assembly, as suggested by previous studies (Uetrecht et al., 2011). Similar tubular assemblies have been reported for other viral proteins, such as SV40, under specific buffer conditions, indicating that buffer composition may influence particle morphology (Palucha et al., 2005; Pistono et al., 2024). At pH 3.0–5.0, GI.3 exhibited both collapsed and hollow particles (Figure 4B). Although the conditions allowing hollow particles to persist remain unclear, the coexistence of different particle forms suggests possible differences in pH stability between *T* = 3 and smaller particles, consistent with previous reports describing distinct stability between *T* = 1 and *T* = 3 forms (Pogan et al., 2020). In contrast to GI.1 and GI.2, GI.4 showed pronounced aggregation under acidic conditions (pH 3.0–5.0), consistent with solubilization assays indicating that GI.4 VP1 produced in the silkworm–BEVS system is highly sensitive to low pH. At pH 9.0, a possible sheet formation was also observed in DLS (Figure 4A), indicating a possible structural transition to tubular forms similar to those observed for GI.2 VP1. Collectively, our stability analyses clearly demonstrate that GI genotypes, particularly GI.4, are intrinsically less robust under varying pH conditions (pH 3.0–5.0) compared to the well-studied GII genotypes (GII.4 or GII.17). This intrinsic instability provides a clear mechanistic basis for the production difficulties encountered previously and highlights the necessity of formulation optimization when developing GI VLP-based vaccines. However, TEM observations were based on ten independent imaging fields, and the number of particles was insufficient to quantitatively assess the ratio of intact to aberrant particles.

Thermal stability is important for determining appropriate storage conditions. We evaluated it by measuring the denaturation temperature of VP1 using DSF. Denaturation midpoints increased in the order of GI.3 < GI.2 < GI.4, all above 70 °C, indicating that NoV VP1 proteins are relatively heat stable. Notably, all GI genotypes that were difficult to produce exhibited melting temperatures exceeding 70 °C. Notably, all GI genotypes that were difficult to produce exhibited melting temperatures exceeding 70 °C. In the DSF analysis, VP1 proteins were purified by SEC prior to measurement, which should be taken into consideration when interpreting these results. Considering that some GI genotypes exhibited low solubility or a tendency toward particle dissociation during extraction and purification, these results indicate that GI VP1 is thermodynamically unstable in the unassembled state but attains high stability upon VLP assembly. Consequently, production-related limitations such as low solubility and particle dissociation may be mitigated by introducing stabilizing mutations to enhance VLP assembly.

While purified VLPs retained PGM-binding activity, their immunogenicity and efficacy as vaccine antigens remain to be determined. Future studies should evaluate their capacity to induce neutralizing antibodies *in vivo* and assess cross-genotype protective efficacy in multivalent formulations. Furthermore, the large-scale production system established in this study yields sufficient quantities of high-quality VLPs for detailed structural characterization and rational antibody engineering. These VLPs also represent safe and versatile surrogate platforms for dissecting virus–host interactions, thereby advancing our mechanistic understanding of NoVs biology.

In conclusion, this study successfully established a genotype-specific purification method for GI norovirus VLPs, overcoming a significant historical barrier to obtaining these critical reagents for vaccine development. Our comprehensive evaluation of particle properties and stability revealed that GI genotypes are intrinsically less stable under varying pH than their GII counterparts, suggesting that production difficulties, such as low solubility and particle dissociation, stem from **VP1 thermodynamic instability in the unassembled state**. These findings provide fundamental data essential for rational VLP formulation and storage. Nevertheless, significant challenges remain, primarily in improving solubility for certain genotypes and validating both the immunogenicity and cross-protective efficacy of the purified VLPs in vivo. Addressing these remaining issues will be indispensable to fully realize the potential of a broad-coverage multivalent VLP-based NoV vaccine.

## Data Availability

The authors acknowledge that the data presented in this study must be deposited and made publicly available in an acceptable repository, prior to publication. Frontiers cannot accept a manuscript that does not adhere to our open data policies.

## Footnotes

1
https://gestwickilab.shinyapps.io/dsfworld/
