# Supplementary material for: Structural and stability differences among GI norovirus virus-like particles produced in silkworm–baculovirus expression vector system
Source: Front Microbiol. 2026 Jan 8;16:1739683. doi: 10.3389/fmicb.2025.1739683 (PMC12823906; doi:10.3389/fmicb.2025.1739683)
Supplement: Supplementary file 1 [file Data_Sheet_1.docx]

Supplementary Material

**Supplementary Figure 1.** **Purification trials of VLPs before and after optimization.**

(A) 10% SDS-PAGE analysis of each fraction collected from the sucrose cushion. Lane numbers correspond to the fraction numbers shown in panel A of Figure 2. The position of VP1 is indicated by an arrow on the right. (B) GI.4 VP1 anion exchange chromatogram obtained using a HiTrap™ Q HP anion exchange column. NaCl concentration is shown in gray, UV absorbance at 260 nm in red, and absorbance at 280 nm in blue. The collection points for the flow-through (FT), wash, and elution fractions are indicated at the top of the graph. Elution was performed using a linear gradient from 0% to 50% NaCl, collecting 19 fractions (5 mL each), followed by 2 fractions with 100% NaCl, yielding a total of 21 fractions (19 from the gradient and 2 from 100% NaCl). (C) 10% SDS-PAGE analysis of fractions corresponding to elution peaks in the chromatogram. Lane numbers correspond to the elution fractions collected as indicated in panel B. The position of VP1 is indicated by an arrow on the right. (D) GI.3 VP1 anion exchange chromatogram obtained using a monolith-type anion exchange column with stepwise NaCl elution. NaCl concentration is shown in gray, UV absorbance at 260 nm in red, and absorbance at 280 nm in blue. The collection points for the flow-through (FT), wash with 15% NaCl, and elution fractions are indicated at the top of the graph. Elution was performed using a step elution with 45% NaCl, collecting 1 mL each. (E) 10% SDS-PAGE analysis of fractions corresponding to elution peaks in the chromatogram. The position of VP1 in the lanes of the collected fractions is indicated by a black box.

**Supplementary Figure 2.** **Changes in particle size at different pH and DSF melting curves.**(A) D50 values measured by dynamic light scattering (DLS) are plotted for each pH. Error bars represent mean ± standard error (SE). (B) Thermal unfolding profiles of VLPs measured by differential scanning fluorimetry (DSF). Representative DSF melting curves are shown, with fluorescence intensity (RFU) monitored as a function of temperature. Each colored line represents an individual VLP sample. Each measurement was performed in triplicate, and the entire experiment was repeated three times (n = 9). The number of each measurement is indicated at the top of the graph. Graphs were generated using the DSFworld web application (https://gestwickilab.shinyapps.io/dsfworld/).

Supplementary Table 1. Results of DLS particle size measurement of purified VP1

| **Genotype** | **Cumulant (nm)** | **Polydispersity indices (PDI)** | **D10 (nm)** | **D50 (nm)** | **D90 (nm)** |
| --- | --- | --- | --- | --- | --- |
| **GⅠ.2** | **39.80 ± 0.35** | **0.08 ± 0.00** | **20.57 ± 0.12** | **24.70 ± 0.12** | **32.67 ± 0.35** |
| **GⅠ.3** | **68.23 ± 0.73** | **0.23 ± 0.01** | **27.27 ± 0.28** | **34.73 ± 1.02** | **47.77 ± 1.62** |
| **GⅠ.4** | **39.50 ± 0.38** | **0.05 ± 0.00** | **22.17 ± 0.62** | **26.83 ± 0.38** | **35.47 ± 0.42** |

Supplementary Table 2. pH stability evaluation of GⅠ.2 VLP using DLS

| **Genotype** | **pH** | **Cumulant (nm)** | **PDI** | **D10 (nm)** | **D50 (nm)** | **D90 (nm)** |
| --- | --- | --- | --- | --- | --- | --- |
| **GI.2** | **3** | **181.73 ± 1.70** | **0.30 ± 0.01** | **27.17 ± 4.51** | **50.50 ± 16.14** | **87.73 ± 19.64** |
|  | **4** | **89.03 ± 0.19** | **0.28 ± 0.00** | **22.63 ± 0.49** | **28.43 ± 1.64** | **40.47 ± 2.27** |
|  | **5** | **76.40 ± 0.17** | **0.27 ± 0.01** | **23.17 ± 1.27** | **29.63 ± 2.71** | **42.83 ± 3.94** |
|  | **6** | **41.80 ± 0.00** | **0.07 ± 0.01** | **21.13 ± 0.35** | **25.83 ± 0.55** | **34.40 ± 0.55** |
|  | **7** | **41.80 ± 0.06** | **0.07 ± 0.01** | **21.40 ± 0.26** | **26.47 ± 0.32** | **35.30 ± 0.40** |
|  | **8** | **41.63 ± 0.09** | **0.06 ± 0.02** | **21.10 ± 0.40** | **25.70 ± 0.69** | **34.13 ± 0.85** |
|  | **9** | **37.23 ± 0.27** | **0.24 ± 0.00** | **15.47 ± 1.33** | **19.07 ± 1.67** | **25.70 ± 1.97** |

Supplementary Table 3. pH stability evaluation of GⅠ.3 VLP using DLS

| **Genotype** | **pH** | **Cumulant (nm)** | **PDI** | **D10 (nm)** | **D50 (nm)** | **D90 (nm)** |
| --- | --- | --- | --- | --- | --- | --- |
| **GⅠ.3** | **3** | **798.47 ± 14.26** | **0.44 ± 0.01** | **147.40 ± 106.69** | **279.60 ± 118.41** | **402.17 ± 164.24** |
|  | **4** | **644.50 ± 23.95** | **0.40 ± 0.01** | **88.30 ± 55.96** | **120.97 ± 78.18** | **362.33 ± 176.23** |
|  | **5** | **512.93 ± 19.65** | **0.33 ± 0.01** | **23.93 ± 2.20** | **29.33 ± 3.09** | **40.80 ± 3.44** |
|  | **6** | **61.23 ± 0.47** | **0.21 ± 0.00** | **23.83 ± 1.13** | **30.47 ± 2.40** | **42.40 ± 3.50** |
|  | **7** | **56.80 ± 0.55** | **0.19 ± 0.01** | **25.30 ± 0.90** | **31.43 ± 1.08** | **42.47 ± 1.32** |
|  | **8** | **58.27 ± 0.18** | **0.17 ± 0.01** | **24.63 ± 1.28** | **30.53 ± 1.59** | **42.03 ± 1.94** |
|  | **9** | **55.07 ± 0.23** | **0.21 ± 0.01** | **21.60 ± 0.75** | **26.40 ± 0.91** | **36.43 ± 1.24** |

Supplementary Table 4. pH stability evaluation of GⅠ.4 VLP using DLS

| **Genotype** | | **pH** | **Cumulant (nm)** | **PDI** | **D10 (nm)** | **D50 (nm)** | **D90 (nm)** |
| --- | --- | --- | --- | --- | --- | --- | --- |
| **GⅠ.4** | **3** | | **2501.20 ± 89.62** | **0.33 ± 0.02** | **1134.93 ± 34.36** | **1381.30 ± 42.03** | **12627.50 ± 10494.57** |
|  | **4** | | **1066.10 ± 27.46** | **0.27 ± 0.01** | **442.10 ± 79.42** | **577.33 ± 112.78** | **806.90 ± 154.70** |
|  | **5** | | **844.10 ± 24.74** | **0.43 ± 0.00** | **39.77 ± 5.93** | **203.40 ± 146.71** | **627.47 ± 298.77** |
|  | **6** | | **42.80 ± 0.17** | **0.07 ± 0.01** | **23.97 ± 0.19** | **29.30 ± 0.15** | **38.70 ± 0.12** |
|  | **7** | | **42.13 ± 0.33** | **0.07 ± 0.00** | **23.63 ± 0.09** | **28.53 ± 0.23** | **37.73 ± 0.47** |
|  | **8** | | **42.20 ± 0.40** | **0.08 ± 0.01** | **22.77 ± 0.88** | **28.37 ± 1.17** | **38.97 ± 2.64** |
|  | **9** | | **58.33 ± 11.44** | **0.12 ± 0.05** | **18.83 ± 1.57** | **22.53 ± 1.42** | **28.93 ± 1.22** |

Supplementary Table 5. GⅠ.2 Evaluation of particle size when changing the pH of VLP from 9 to 7

| **Cumulant (nm)** | **PDI** | **D10 (nm)** | **D50 (nm)** | **D90 (nm)** |
| --- | --- | --- | --- | --- |
| **50.5 ± 1.29** | **0.11 ± 0.01** | **19.47 ± 0.47** | **23.07 ± 0.42** | **29.23 ± 0.44** |
